# Flavivirus replication kinetics in early-term placental cell lines with different differentiation pathways

**DOI:** 10.1186/s12985-021-01720-y

**Published:** 2021-12-14

**Authors:** Julio Carrera, Alice M. Trenerry, Cameron P. Simmons, Jason M. Mackenzie

**Affiliations:** 1 Department of Microbiology and Immunology, University of Melbourne, Peter Doherty for Infection and Immunity, Parkville, Melbourne, VIC 3010 Australia; 2 Institute of Vector-Borne Diseases, Monash University, Clayton, VIC 3800 Australia

**Keywords:** West Nile virus, Zika virus, Flavivirus replication, Placenta

## Abstract

**Background:**

The uncontrollable spread of Zika virus (ZIKV) in the Americas during 2015–2017, and its causal link to microcephaly in newborns and Guillain-Barré syndrome in adults, led the World Health Organisation to declare it a global public health emergency. One of the most notable features of ZIKV pathogenesis was the ability of the virus to pass the placental barrier to infect the growing foetus. This pathogenic trait had not been observed previously for medically important flaviviruses, including dengue and yellow fever viruses.

**Methods:**

In this study we evaluated the replication kinetics of ZIKV and the related encephalitic flavivirus West Nile strain Kunjin virus (WNV_KUN_) in early-term placental cell lines.

**Results:**

We have observed that WNV_KUN_ in fact replicates with a greater rate and to higher titres that ZIKV in these cell lines.

**Conclusions:**

These results would indicate the potential for all flaviviruses to replicate in placental tissue but it is the ability to cross the placenta itself that is the restrictive factor in the clinical progression and presentation of congenital Zika syndrome.

## Background

During the 2016 Zika virus (ZIKV) epidemic one notable feature of the pathogenesis in pregnant mothers was the ability of ZIKV to pass the placental barrier. As infection progressed it was shown that ZIKV had a distinctive tropism for the central nervous system (CNS) of the developing embryo, which led to microcephaly, an extreme manifestation of the now recognized congenital Zika syndrome. This new teratogenic syndrome includes a full range of other neurological complications such as spasticity, seizures, brainstem disfunction, cortical calcifications and malformations, brainstem/cerebellar hypoplasia, ventriculomegaly, visual disturbances and optic nerve abnormalities [1]. Virions that come into contact with the placental-foetal interface, lead to the infection of the foetus neuronal cells [2, 3], which show dysregulated cell replication cycles, reduced cell division, augmented cell death and breaking of double stranded DNA, which in turn inhibits DNA repair [2, 4]. Pre- and peri-implantation ZIKV infection also impairs foetal development and increases the risk of spontaneous abortion by targeting trophectoderm cells even earlier in the development during the pre-placental stage [5].

The transfer of ZIKV to the foetus through the placenta occurs after the colonization of the decidua, whose cells are permissive to the infection of several viruses including ZIKV. Viral particles then migrate to extravillious trophoblasts and cytotrophoblasts in a process more likely to happen during the first trimester of gestation, as syncytiotrophoblasts appear resistant to infection throughout pregnancy [6, 7]. Indeed, the most severe cases of foetal damage have been shown to occur when the infection happens during the first trimester of pregnancy with symptoms including chronic villitis, edema, trophoblastic lesions and increase in HBCs in contrast to third trimester placentas, which showed only delayed villous maturation with limited pathological changes [8].

It has been proposed that trans placental transmission of the virus without disruption of the placental barrier might be also possible, caused by antibodies that bind ZIKV with low affinity such as those generated by previous infections with other *Flavivirus* such as dengue virus (DENV) [6]. Many models for the study of the placenta exist, from primary cells to trophoblastic cell lines such as HTR8/SVneo, BeWo, JEG and JAR [9]. HTR8/SVneo and BeWo cells are both trophoblastic cell lines originated from a first trimester placenta; BeWo cells are a representative of the villous pathway that remains within the placenta and HTR8/SVneo cells are a representative of the extravillious pathway, which migrate and infiltrate into the maternal decidua where they play important functions by interacting with cells in the stroma and modify blood vessels [10]. ZIKV is the first *Flavivirus* known to cause damages at this scale in the developing fetus, and the fact that only 20% of the infected population show any symptoms, makes this virus and the mechanisms that allow it to bypass the normally strict placental protection very important to understand. Moreover, it becomes imperative to understand other flaviviruses such as DENV or West Nile virus (WNV) from this point of view, since mutations in these viruses could potentially confer ZIKV characteristics to other flaviviruses.

For these reasons we aimed to describe the replication kinetics of WNV strain Kunjin virus (WNV_KUN_) and ZIKV in two early-term placental cell lines with different differentiation pathways to gain insight whether other or all flaviviruses could also potentially infect placental tissue and to what degree. It is equally important to understand how different populations of early-term placental cells could affect the progression of uterine/placental ZIKV infection. Understanding these dynamics could lead us to find more profound answers of why ZIKV is capable of crossing the placenta and other viruses can’t or why if both are capable of infecting this type of tissue, only ZIKV dramatically affects the embryo development.

## Materials and methods

### Viruses and cell lines

The ZIKV-Asian used in this report was originally donated by the Victorian Infectious Diseases Reference Laboratory (VIDRL) and corresponds to the Asian-Pacific clade (GenBank: KX806557.3); the virus corresponds to a third passage and as propagated in Vero cells. WNV_KUN_ MRM61C was also propagated to a third passage in Vero cells.

Vero C1008 (African Green Monkey Kidney), were maintained in Dulbecco’s Modified Eagle Medium (DMEM) (Gibco™ Catalog number: 11995073) supplemented with 10% fetal bovine serum (FBS) and 1% Glutamax (Gibco™ Catalog number: 35050061) unless differently stated. HTR8/SVneo cells (*Homo sapiens* first trimester placenta) were maintained in RPMI 1640 (GibcoTM Cat. Number 22400-071) supplemented with 5% foetal bovine serum (FBS) and 1% NEAA (GibcoTM Cat. Number 11140050). BeWo cells (*Homo sapiens* first trimester placenta) were maintained in DMEM/F12 1640 (GibcoTM Cat. Number 11320033) supplemented with 10% foetal bovine serum (FBS) and 1% NEAA (GibcoTM Cat. Number 11140050).

### Plaque assay

Vero cells were seeded into 12 well plates to reach 80% confluency next day (1 × 10^5^ cells per well). Virus was serially diluted tenfold in DMEM without FBS and cells were infected for 2 h at 37 °C. After the incubation period, 2 mL of 2% FBS/DMEM/low melting point agarose overlay was added. Cells were further incubated for (a) 48 h for WNV_KUN_ and (b) 96 h for ZIKV. Cells were fixed for 1 h with 10% formalin solution and the overlay was then removed and cells were washed and stained with 0.1% Toluidine blue in ddH_2_O for 1 h. Wells were rinsed with water and the numbers of visible plaques counted. The experiment was completed each time in duplicates and the average number of the plaques at the highest dilution was used to calculate the viral titre as plaque forming units per mL (PFU/mL) using the following equation:

$${\text{PFU}}/{\text{mL}} = (\# \;{\text{of plaques}} \times {\text{dilution factor}})/{\text{volume plated}}$$

### Infection of Vero, HTR8/SVneo and BeWo cells

Cells were seeded the day before infection in 6 well plates to reach a confluency of 80% (1 × 10^5^ for Vero cells and 2 × 10^5^ for BeWo and HTR8/SVneo cells). Cells were then infected with previously titrated in Vero cells ZIKV and WNV_KUN_ at a multiplicity of infection (MOI) of 1. Total RNA, cell lysates and released virus was collected 12, 24, 48, 72 and 96 h.p.i. Results were extracted from 3 independent experiments.

### RNA extraction and ZIKV quantitative real-time polymerase chain reaction (qRT-PCR)

Total RNA was isolated and DNAse treated using the RNeasy kit (Qiagen Cat. Number 74106). cDNA was synthesized using the SuperScript® III First-Strand Synthesis System (Invitrogen™) with random hexamers. For ZIKV and WNV_KUN_ quantification, cDNA was amplified by real-time qPCR (BioRad® CFX96 Touch Real-Time PCR Detection System), with 1 cycle at 50 °C for 10 min, 1 cycle at 95 °C for 30 s, followed by 45 amplification cycles of 95 °C for 3 s, 60 °C for 30 s, 72 °C for 1 s, and a final cooling cycle of 4 °C for 1 s using the iTaq Universal SYBR Green Supermix (BioRad® Cat. Number 1725120). The relative ZIKV and WNV_KUN_ copies were determined by normalizing with the reference GAPDH (Glyceraldehyde 3-phosphate dehydrogenase) gene. Primers used are listed below (Table 1).

**Table 1:** List of primers and primer sequences used for ZIKV, WNV_KUN_ and GAPDH RNA detection by qRT-PCR

Primers	Sequence		Reference
Zika4481-forward	5′–CTGTGGCATGAACCCAATAG–3′		[11]
Zika4552c-reverse	5′–ATCCCAKAGRGCACCACTCC–3′		
WNV_KUN_-forward	5′–TCAAGAATAACTTGGCGATCCA–3′		[12]
WNV_KUN_-reverse	5′–TCACCTAGGACCGCCCTTT–3′		
GAPDH-forward	5′–ACAGTCCATGCCATCACTGCC–3′		[12]
GAPDH-reverse	5′–GCCTGCTTCACCACCTTCTTG–3′		

### SDS-PAGE and western blot analysis

Cells were lysed with NP-40 lysis buffer containing protease inhibitors, incubated in constant rotation at 4 °C during 30 min and centrifuged at 15800*g* and 4 °C for 30 min to pellet cell debris; the supernatant was then stored at – 80 °C for further analyses. 4 × Laemmli loading buffer was added to an aliquot of the sample (3:1) and samples were boiled for 5 min at 95 °C and put on ice. Lysates were loaded onto a 4–12% Tris–Glycine polyacrylamide gel and proteins were separated at 120 V for 90 min in Tris–glycine buffer and transferred to a nitrocellulose membrane at 100 V for 60 min in Western blot transfer buffer. The nitrocellulose membrane was blocked overnight in 5% BSA in phosphate-buffered saline (PBS). Primary antibodies incubated with the membrane for 4 h at room temperature (RT) on a rotator in blocking buffer. Following, the membranes were washed four times with PBS-T for 5 min. Secondary antibodies were then diluted in blocking buffer and incubated on rotator for 60 min at RT. The membrane was then washed twice in PBS-T and twice in PBS for 15 min each time. Blots were visualized using a BioRad® PharoFX scanner. Precision Plus Protein™ Protein Standard-Bio-Rad® was used as reference for expected molecular weight estimation.

### Flow cytometry

Cells were infected with WNV_KUN_ or ZIKA at an M.O.I. of 1 for 48 h before harvesting and incubation in a Live/dead stain (eBioscience™ Fixable Viability Dye eFluor™ 780). The cells were then fixed with 4% PF/PBS and permeabilised with 0.5% T × 100 in PBS. The cells were then incubated with anti-E antibodies (clone 4G2) for 1 h at 4 °C and then with the secondary antibodies (Alexa-Flour 488) for 45 min at 4 °C. Cells were subsequently washed in staining buffer, resuspended in PBS, and kept in the dark at 4 °C until flow cytometry analysis. Flow cytometry data were collected with a BD LSRFortessa analyser using BD FACSDiva software (BD Biosciences). Data were analysed using FlowJo analysis software and graphs were assembled using Prism 9 software and significance from replicate experiments determined via RM one-way ANOVA. *p < 0.05 and ****p < 0.0001.

### Immunofluorescence analysis (IFA) and confocal microscopy

Coverslips with infected cells (Vero, HTR8/SVneo and BeWo cells) were incubated in primary antibodies specific to protein of interest diluted in 25 µl PBS/1% bovine serum albumin (BSA) for 1H at RT, then washed 3 times in PBS/0.1% BSA for 5 min each time and incubated in secondary antibodies diluted in 25 µl PBS/0.1% BSA for 45 min at RT. Coverslips were then washed twice with PBS and left for 5 min in 300 µl of 4′,6-diamidino-2-phenylindole (DAPI) stain in PBS for nuclei staining. Coverslips were then rinsed twice with PBS and twice with deionized MilliQ water for further mounting onto microscope slides using Ultramount mounting media (Fronine) and left to dry 1H/overnight at RT. Slides were then stored at 4 °C and cells were analyzed using a Zeiss 710 Confocal Microscope and ZenTM Zeiss ® software.

## Results

### Both West Nile and Zika virus infection of cells of placental origin release high titres of infectious virus

**Fig. 1:**
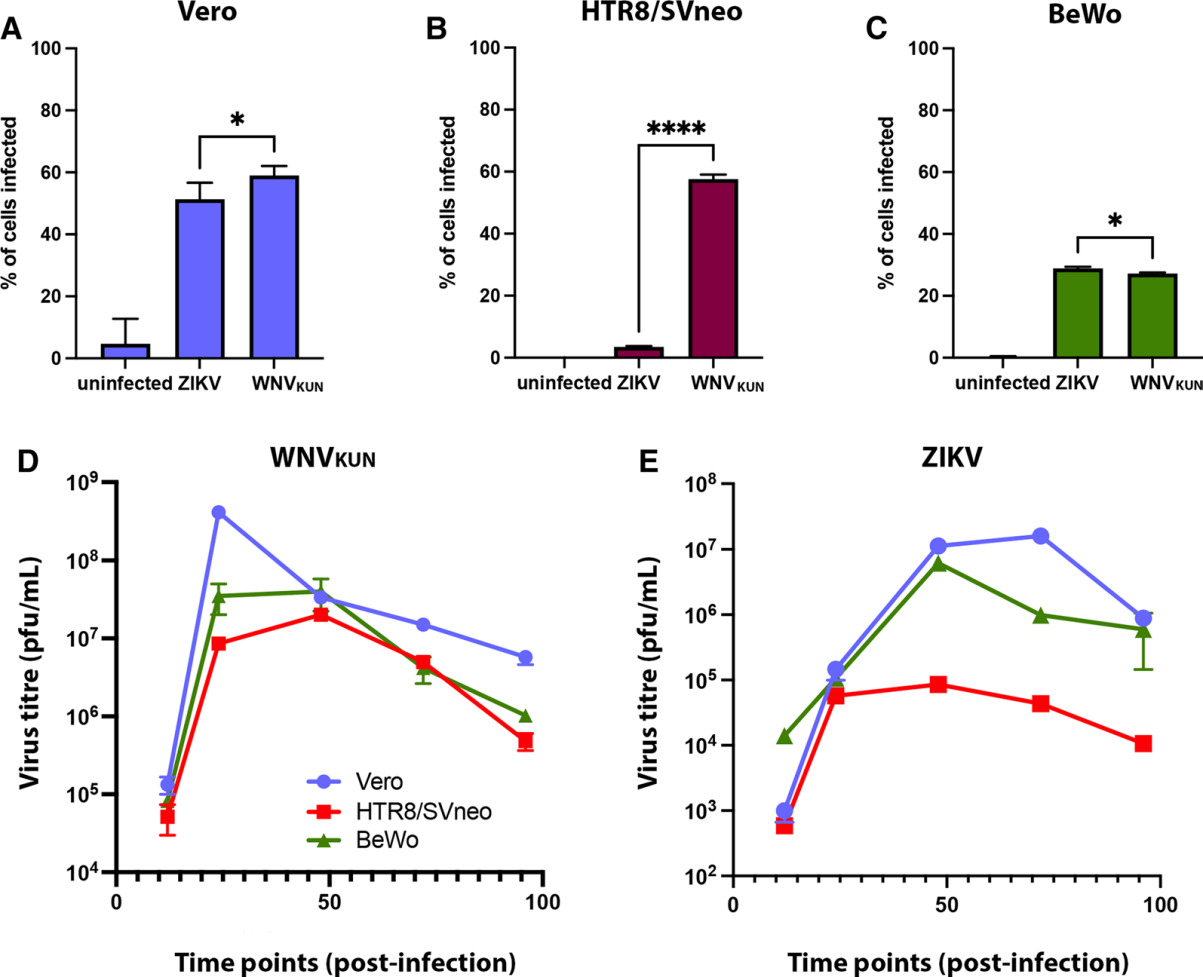
Replication and production of infectious virus after ZIKV and WNV_KUN_ infection of Vero, HTR8/SVneo and BeWo cells. Infection rates of the two viruses on the different cell lines was initially assessed by Flow Cytometry. Basically Vero (**A**), HTR8/SVneo (**B**) and BeWo cells (**C**) were infected with either WNV_KUN_ or ZIKV at an M.O.I. of 1 and supernatants were collected at 48 h post-infection. Infected cells were identified with anti-E (4G2) antibodies and AF488. Analysis was performed on a BD LSRFortessa analyser. For the virus release: cells were infected with either WNV_KUN_ (**D**) or ZIKV (**E**) at an M.O.I. of 1 and supernatants were collected at various time post-infection, namely 12, 24, 48, 72 and 96 h.p.i.. Infectious virus release was determined by plaques assay on Vero cells. Error bars: ± SEM, n = 3, *p < 0.05; ****p < 0.001

To evaluate the replication of WNV_KUN_ and ZIKV in the different placental cell lines we initially assessed the infect rates by FACS analysis (Fig. 1A–C). Vero, HTR8/Svneo and BeWo cells were infected with each virus at an MOI of 1 and the percentage of infected cells was determined at 48 h.p.i. by immunostaining with anti-E antibodies. As can be observed Vero cells were infected at a high rate of ~ 50–60% by both viruses and BeWo cells at ~ 30% for both viruses (Fig. 1A and C). Strikingly, the HTR8/Svneo cells showed a large difference with WNV_KUN_ infecting these cells at ~ 60% but ZIKV only infecting at ~ 5% (Fig. 1B).

To compare and contrast the replication kinetics of WNV_KUN_ and ZIKV in the different cell lines we initially determined the recovery of infectious virus over the time course of the experiment, namely 12, 24, 48, 72 and 96 h.p.i. All cell lines were infected with either virus at a MOI of 1 and secreted infective extracellular ZIKV and WNV_KUN_ was measured by plaque assay at 5 different time points in 3 independent experiments. In Vero cells, ZIKV reaches its peak in virus titre at 72 h.p.i. while in contrast WNV_KUN_ starts declining after only 24 h.p.i., indicating that these two viruses have a different replication kinetics in this cell line (Fig. 1D). The kinetics of replication in the BeWo cells was observed to be similar to that in Veros, although both viruses reached a peak at 48 h.p.i. and WNV_KUN_ infection produced ~ 1 log higher titre of infectious virus. The most striking difference was observed in the HTR8/Svneo cells, where a different pattern was observed such that WNV_KUN_ replicated as robustly as in the BeWo cells. However, ZIKV replication was severely attenuated in the HTR8/Svneo cells with a reduction of 2–3 logs observed between these cells and the BeWo and Vero cells (Fig. 1E).

These results make two intriguingly observations: (1) WNV_KUN_ appears to replicate more robustly in the placental cell lines than ZIKV, and (2) the release of infectious ZIKV is severely attenuated in the HTR8/Svneo cells but not BeWo cells. Interestingly, WNV_KUN_, which is a virus not commonly related to microcephaly or other congenital disorders but is encephalitic, replicates highly in both placental cell lines.

### West Nile and Zika viruses have differing efficiency in replicating and producing viral RNA in HTR8/Svneo and BeWo cells

**Fig. 2:**
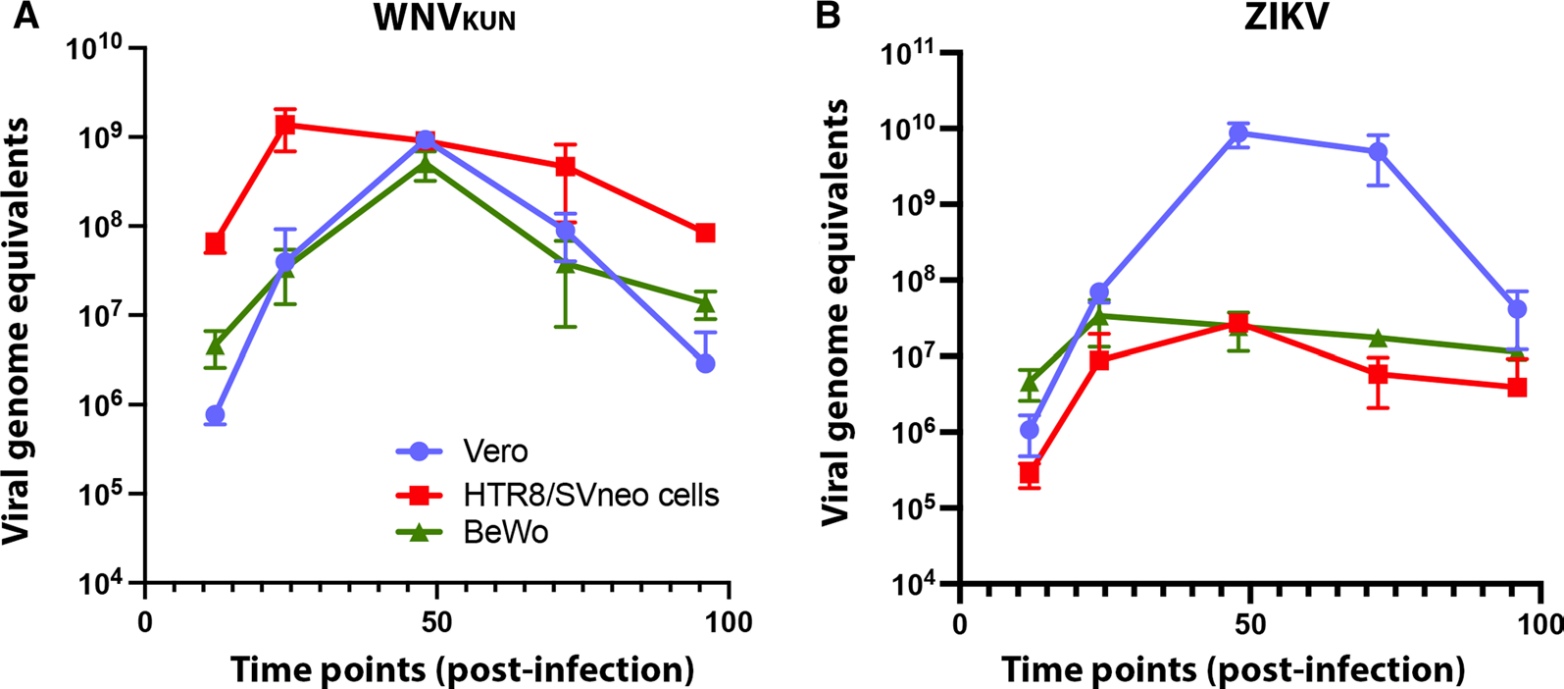
ZIKV and WNV_KUN_ genome equivalents in Vero, HTR8/SVneo and BeWo cells. Cells were infected with WNV_KUN_ (**A**) or ZIKV (**B**) at an M.O.I. of 1, and virus genome replication was analysed by qRT-PCR at various time post-infection, namely 12, 24, 48, 72 and 96 h.p.i.. Error bars: ± SEM, n = 3

Due to the observed differences in the production and release of infectious ZIKV and WNV_KUN_ virus particles, we aimed to evaluate at which stage of the replication cycle contributed to these differences. Thus, we first investigated the rate at which both viruses generated genomic RNA in the different cell lines (Fig. 2). We observed that the replication of ZIKV RNA was attenuated in the placental cell lines compared to Vero cells (Fig. 2B). However, ZIKV RNA replication reached a peak at 48 h.p.i. whereupon RNA copies of the genome decrease very slowly over time. Interestingly though we observed that the restricted production of viral genomes in the HTR8/Svneo cells infected with ZIKV correlated with the low levels of recovered infectious virus. Intriguingly though this restriction in viral genome amplification was also observed in the BeWo cells which were high producers of infectious ZIKV. Again, WNV_KUN_ genome replication was observed to be robust in all cell lines, but we did observe that once RNA replication reached a peak at 48 h.p.i. the copies of the viral genome also decreased in time but at a more accelerated rate (Fig. 2A). Cell death was less evident in both placental cell lines with both viruses than in Vero cells, something that was especially remarkable with WNV_KUN_, which is known to rapidly kill most of the cells within only 48 h.p.i.

This results again highlight the ability of WNV_KUN_ to replicate effectively in the placental cell lines and the attenuated replication of ZIKV in the HTR8/Svneo cells. It would appear that although the production of viral RNA is low in the ZIKV-infected cells this is still effectively used in generating infectious virus.

### Viral protein expression in HTR8/Svneo and BeWo cells reflects the production of viral RNA

**Fig. 3:**
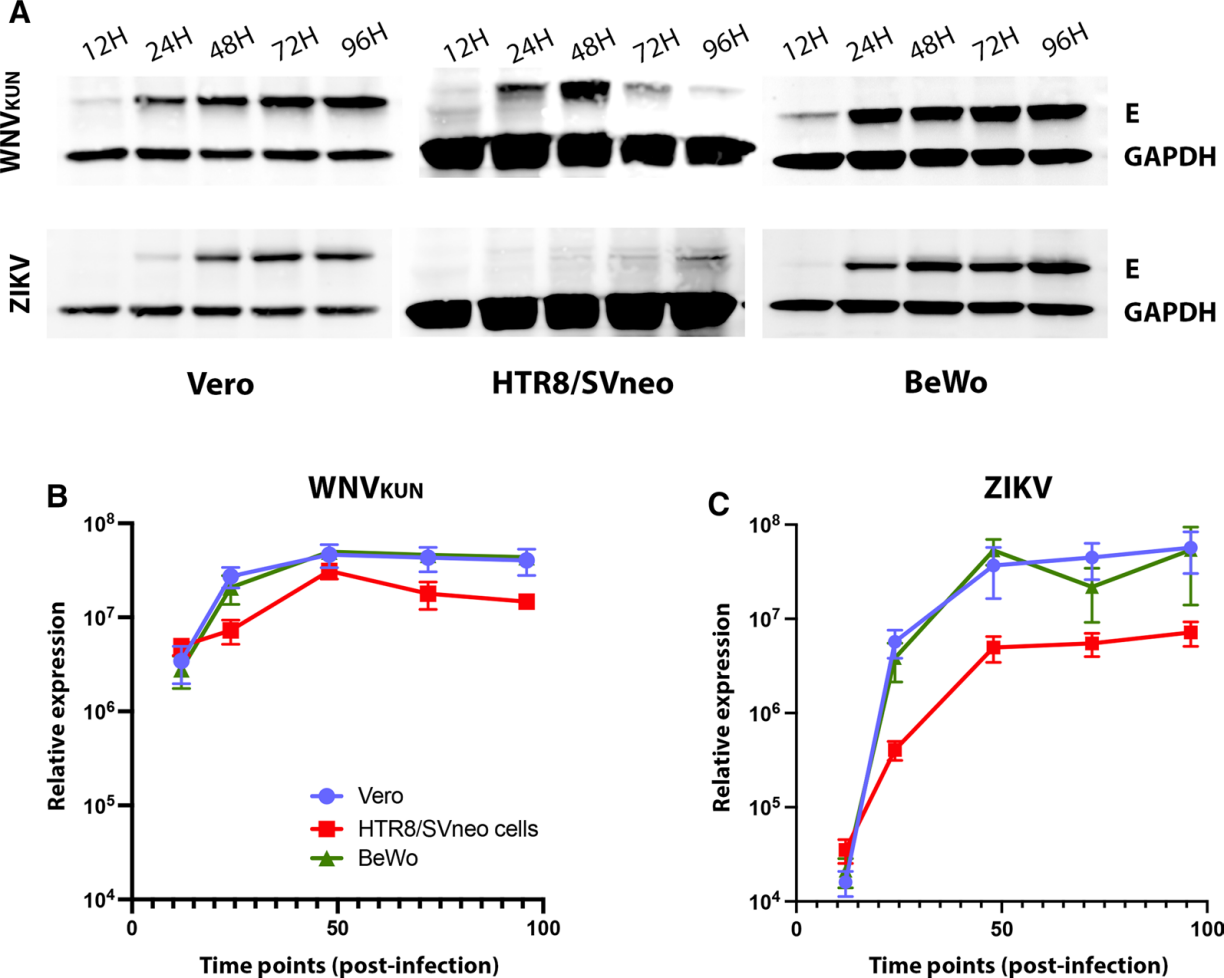
ZIKV and WNV_KUN_ viral protein expression in Vero, HTR8/SVneo and BeWo cells. **A** Production of the viral envelope protein (E) was assessed by western blot after lysates from WNV_KUN_ or ZIKV-infected cells at various times post-infection, namely 12, 24, 48, 72 and 96 h.p.i.. The relative viral protein was compared to the cellular protein GAPDH and was quantified in **B** and **C**, for WNV_KUN_ or ZIKV respectively. Error bars: ± SEM, n = 3

We observed above that ZIKV and WNV_KUN_ replication were efficient in Vero and BeWo cells, but that ZIKV replication was attenuated in HTR8/Svneo cells. To correlate the RNA and infectious virus analyses we investigated the production and expression of the viral envelope protein over the course of infection, namely 12, 24, 48, 72 and 96 h p.i. (Fig. 3). Flaviviruses hijack the host translation system to produce structural and non-structural viral proteins, which are necessary for the production of new virions and to control the host defence systems against viral infections.

WNV_KUN_ and ZIKV, produced comparable levels of the envelope I protein in both Vero cells and BeWo placental cells with both viruses showing a peak around 48 h.p.i. (Fig. 3A). The detectable amounts of Envelope protein with WNV_KUN_ declines in all cases after 48 h.p.i., which is evidenced by the slightly higher cell death in comparison with ZIKV, from which protein is in most cases is still accumulating even at 96 h.p.i. (Fig. 3A and B). This can also be reflected in the utilization of the E protein in producing infectious virus (Fig. 1D and E). Again, the most significant observation is the robust production of WNV_KUN_ E protein in the HTR8/SVneo cells compared to the almost undetectable amount of ZIKV E protein. The lower but sustained presence of ZIKV in HTR8/SVneo placental cells by all the methods of detection here described, could potentially point to an inefficient activation of signaling immune pathways against this viral infection and in turn. Alternatively, it could reflect the biophysical properties of these cells in cell differentiation, however it should be noted that although ZIKV replication is restricted in this cell type WNV_KUN_ could still replicate very effectively.

### Expression and distribution of the ZIKV and WNV_KUN_ envelope protein in infected BeWo and HTR8/SVneo cells

**Fig. 4:**
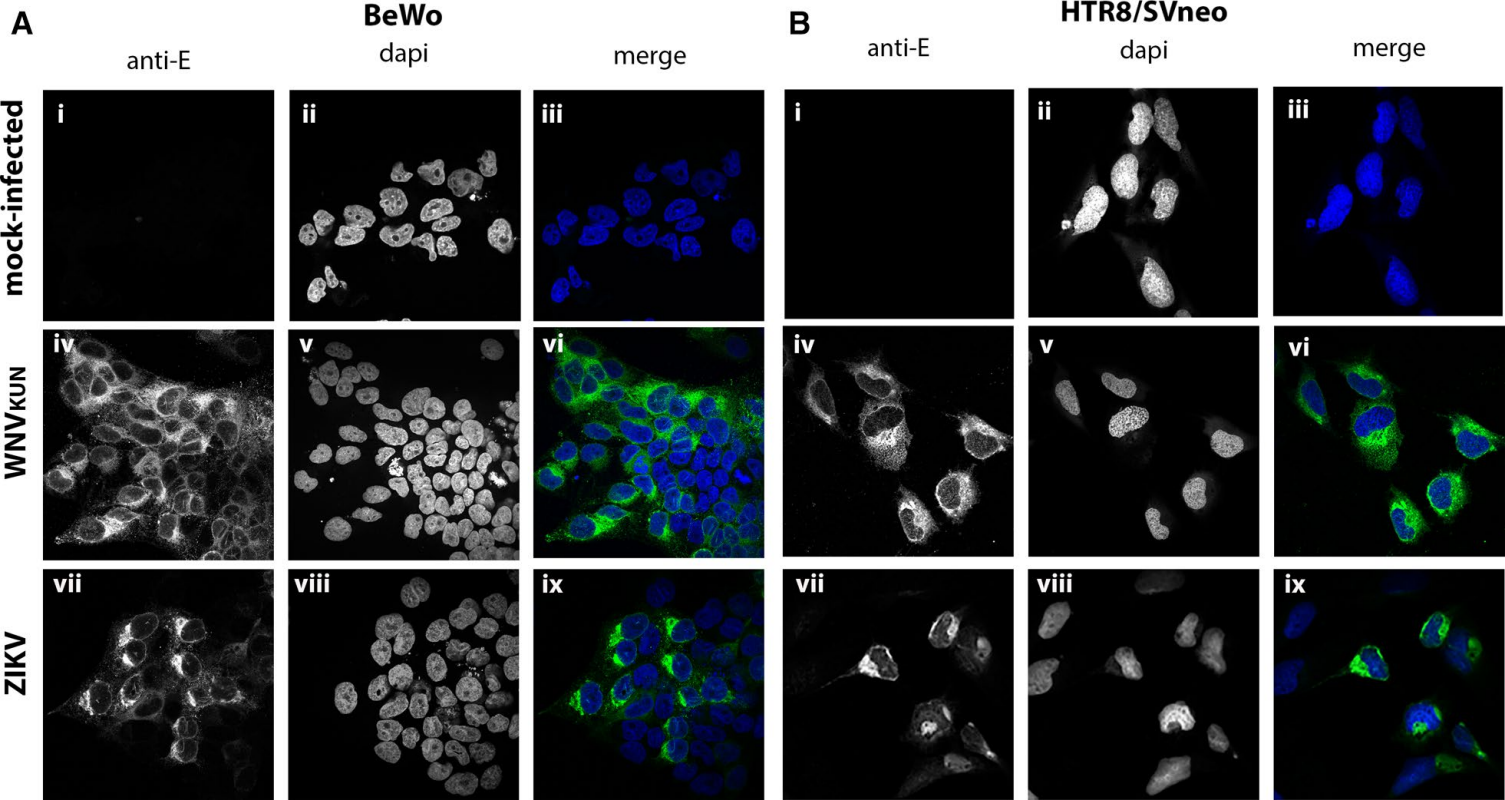
ZIKV and WNV_KUN_ viral protein localisation in HTR8/SVneo and BeWo cells. Viral envelope protein (E) expression and distribution was assessed by IFA at 48 h.p.i. in BeWo (**A**) or HTR8/SVneo (**B**) cells. Mock-infected (panels **Ai-iii** and **Bi-iii),** WNV_KUN_ (panels **Aiv-vi** and **Biv-vi**) and ZIKV (panels **Avii-ix** and **Bvii-ix**) E expression was visualised with anti-envelope antibodies and species-specific IgG conjugated to AF488 (green; panels **Ai, iv, vii** and **Bi, iv, vii**), and the nuclei were counterstained with dapi (blue; panels **Aii, v, vii** and **Bii, v, vii**). Merged images are in the right-hand panels (**Aiii, vi, ix** and **Biii, vi, ix**) as indicated. Images were processed and assembled in Adobe Photoshop

To further characterise the infection of the different cell lines we utilised IFA to identify any differences in the localisation and distribution of the enveloI(E) protein in the HTR8/SVneo and BeWo cell lines. Cells were infected with either virus at an m.o.i. of 1 and at 48 h.p.i. the cells were fixed and processed for immunofluorescence analysis with a monoclonal anti-E antibody that was cross-reactive for both WNV_KUN_ and ZIKV (Fig. 4). We observed that for both viruses in both cell lines there was efficient and effective protein production localised primarily in the perinuclear region but also dispersed within the cytoplasm. The only difference was the lower infection rate of ZIKV in the HTR8/SVneo cells compared to BeWo (compare ***Aiv–vi*** with ***Biv–vi***). WNV_KUN_ appeared to infect both cell lines with equal efficiency (***Ai–iii*** with ***Bi–iii***).

Overall, this data supports our previous analyses indicating that WNV_KUN_ can effectively infect and replicate within both placental cell lines whereas ZIKV appears to infect the BeWo cells to a greater level that the HTR8/SVneo cells.

## Discussion

The placenta is a temporary foetal organ that develops from the blastocyst shortly after embryo implantation in placental mammals. The placenta is the interphase through which important functions of nutrient, gas and waste exchange happen between the mother and the foetus, in addition to playing important endocrine functions to regulate maternal and foetal physiology during pregnancy. The placenta connects the mother to the developing embryo via the umbilical cord [13].

It is now well-established that ZIKV can infect placental tissue and can cross this barrier to infect the developing baby in humans [14] and in animal models of infection. This can lead to the Zika congenital syndrome, which besides its most important feature microcephaly, can also exhibit dyskinetic signs, corticospinal alterations, neuromuscular damage or a mix of all these signs [15]. Some limited reports have indicated the WNV and DENV can indeed infect placental tissue with an associated increase in the severity of disease in both the mother and the foetus in humans and mice [16, 17], but ZIKV is the first flavivirus known to cause such detrimental effects in the development of the CNS of newborns. First trimester maternal ZIKV infection has proved to be an important risk factor for a more severe spectrum of congenital Zika syndrome including microcephaly [18].

Here we analysed two cell lines that are first-trimester trophoblast cell models, HTR8/SVneo and BeWo cells, in addition to Vero cells, which are a well-established cell model for the study of flaviviruses. HTR8/SVneo cells and BeWo are representatives of two very distinct differentiation pathways of trophoblastic cells: BeWo cells are representatives of the villious pathway that remains in the outermost level of the placenta and can fuse to form the syncytiotrophoblast, secreting chorionic gonadotropin, which is responsible for the maternal recognition of pregnancy; HTR8/SVneo cells on the other hand, are representatives of the extravillious pathway, that migrate and infiltrate in the decidua, where they participate in arterial remodeling and invasion of veins, lymphatic and glands [19]. Based on these characteristics, any detrimental effect on BeWo cells may predict impairment of placental growth, while any effect towards HTR8/SVneo cells may explain unsatisfactory blastocyst implantation and placentation in vivo [10].

In this report we observed that both ZIKV and WNV_KUN_ replicate effectively in the villous trophoblast-like cell line BeWo, although the kinetics of replication were slightly different. Significantly, our observations indicate that ZIKV in contrast to WNV_KUN_ replicates less efficiently in the extravillous trophoblast-like HTR8/SVneo cells. This may explain that ZIKV may be less likely to compromise blastocyst attachment when the infection takes place as a pre- or peri- implantation event before placentation [5]. Upon infection of the mother, ZIKV can pass through the decidua and the early placenta to the embryo. This is a process where placentation and embryo development can be affected, even leading to miscarriage and resorption in mice [5, 20], but where the embryo attachment itself is less compromised. In contrast, WNV_KUN_ could potentially cause more spontaneous miscarriages if the infection is pre- or peri-implantation, since it was able to replicate much more efficiently not only in BeWo cells, but also in HTR8/SVneo cells (Figs. 1, 2 and 3).

After implantation, when villous trophoblasts differentiate into syncytiotrophoblasts the story could be the opposite, since experiments in mice dams infected with WNV at 11.5- and 14.5-days post coitus (dpc) resulted in little to no foetal infection [21], while mice infected with ZIKV at embryonic day 17.5 showed embryonic resorption (demise), growth limitations, malformations and anatomical developmental defects consistent with ZIKV-associated foetal outcomes [20]. What these studies also imply is that the impact and progression of disease is not entirely about infecting cells within the placenta but rather the accessibility of the different flaviviruses to the placenta and the tissues within. It is thus apparent that ZIKV can cross the placenta effectively, but WNV cannot, although if this did happen WNV could replicate as efficiently potentially causes similar outcomes to ZIKV. Nonetheless, WNV and other flaviviruses such as DENV or Yellow Fever virus are not commonly studied from the gestational point of view and therefore little conclusions can be drawn at this point, although small epidemiological studies have found events of miscarriage among WNV cases in the United States [22]. Among flaviviruses, only the outbreak of the magnitude of Zika between 2016 and 2018 allowed the study of implications of flavivirus infections in pregnancy and embryo development in such a large scale. Consequently, it remains critically important to include these questions in new protocols of DENV and other arbovirus surveillance.

In discussing these results, we must acknowledge a few caveats to the study; (1) we cannot discount the possibility that the immortalised placenta cells used in this study maybe inherently different to primary tissue. Thus, some tissue culture adaptations may have occurred to allow efficient replication of WNV_KUN_ versus ZIKV. Notably a recent article has evaluated a number of placental cell lines and showed different properties depending on the functional attribute assessed [23]. (2) We are comparing the replication kinetics of two different viruses so we cannot directly relate efficient replication with disease. Essentially, our aim was not to do this but rather to determine if the replication of WNV_KUN_ in the placental cell lines was altered or inhibited to ascertain whether susceptibility and/or permissibility could contribute to pathology. This does appear to be the case though. (3) Currently, we do not understand the factors that contribute to the ability of ZIKV to infect placental tissue in contrast to WNV_KUN_. It is evident that this is multifactorial involving cell surface receptor usage, cellular signalling events, cellular responses and the role of the immune response [24–26]. This will be the target of future studies to fully appreciate the tropism and disease determinants of one flavivirus over the other, to additionally provide targets for the antiviral therapeutic development.

## Conclusions

Our results have shown that all flaviviruses may have the potential to replicate in placental tissue but it is the ability to cross the placenta itself that is the restrictive factor in the clinical progression and presentation of congenital Zika syndrome. This is important when considering the pathogenesis of flavivirus diseases and the expanding clinical outcomes associated with infection.

## Data Availability

All data generated or analysed during this study are included in this published article.

## Abbreviations

ZIKV: Zika virus
WNV_KUN_: West Nile virus strain Kunjin virus
CNS: Central nervous system
DENV: Dengue virus
DMEM: Dulbecco’s modified eagle medium
BSA: Bovine serum albumin
MOI: Multiplicity of infection
h.p.i.: Hours post-infection
FBS: Foetal bovine serum
qRT-PCR: Quantitative real-time polymerase chain reaction
DAPI: 4′,6-Diamidino-2-phenylindole
RT: Room temperature
PBS: Phosphate-buffered saline
E: Envelope
